# Directional Preference in Avian Midbrain Saliency Computing Nucleus Reflects a Well-Designed Receptive Field Structure

**DOI:** 10.3390/ani12091143

**Published:** 2022-04-28

**Authors:** Jiangtao Wang, Longlong Qian, Songwei Wang, Li Shi, Zhizhong Wang

**Affiliations:** 1 Henan Key Laboratory of Brain Science and Brain-Computer Interface Technology, School of Electrical Engineering, Zhengzhou University, Zhengzhou 450001, China; zzuwjt@163.com (J.W.); zdhqll@163.com (L.Q.); wangsongwei@zzu.edu.cn (S.W.); 2 Department of Automation, Tsinghua University, Beijing 100084, China

**Keywords:** pigeon, isthmi pars magnocellularis, motion direction, computational model, biological plausibility

## Abstract

**Simple Summary:**

Directional preference neurons has been found in many vertebrate sensory systems. The isthmi pars magnocellularis (Imc) in avian midbrain, playing a key role in visual selective attention, shows impressive motion directional preference, but little is known about the physiological basis of this phenomenon. Herein, artificial visual stimuli, statistical analyses, and a neural computational model were used to unravel this mystery. This study deepens the understanding of the relationship between the directional preference and special receptive field structure of pigeon’s (*Columba livia*) Imc neuron.

**Abstract:**

Neurons responding sensitively to motions in several rather than all directions have been identified in many sensory systems. Although this directional preference has been demonstrated by previous studies to exist in the isthmi pars magnocellularis (Imc) of pigeon (*Columba livia*), which plays a key role in the midbrain saliency computing network, the dynamic response characteristics and the physiological basis underlying this phenomenon are unclear. Herein, dots moving in 16 directions and a biologically plausible computational model were used. We found that pigeon Imc’s significant responses for objects moving in preferred directions benefit the long response duration and high instantaneous firing rate. Furthermore, the receptive field structures predicted by a computational model, which captures the actual directional tuning curves, agree with the real data collected from population Imc units. These results suggested that directional preference in Imc may be internally prebuilt by elongating the vertical axis of the receptive field, making predators attack from the dorsal-ventral direction and conspecifics flying away in the ventral-dorsal direction, more salient for avians, which is of great ecological and physiological significance for survival.

## 1. Introduction

The predator–prey interaction is responsible for the observed changes in abundance of the urban-sensitive bird group. No matter whether they are in urban or rural areas, for birds living under high predation risk, maintaining an on-going monitoring of predators is necessary for survival [1,2]. Moreover, the flock size of different bird species may influence how species respond to perceived threats, and gregarious birds initiate flight after detecting a potential predator earlier (longer flight initiation distance) when aggregated in large flocks. Clearly, this higher vigilance benefits from many eyes scanning for birds, and increases the possibility of predators to be detected early [3]. Therefore, the ability of focusing on objects moving in the d-v (dorsal-ventral) direction (may mean an aerial predator) as well as remaining vigilant against other conspecifics flying away in the v-d (ventral-dorsal) direction (may mean a tangential approaching potential threat, e.g., human, cat) in visual space is important for avians’ survival. These targets are always salient within the surroundings, and can automatically capture the animal’s attention in a bottom-up style [4]. However, the physiological basis underlying this phenomenon is unclear, and a better understanding of avian visual perception will enhance our understanding of escape in birds and enhance our ability to manage it by designing specific stimuli that best capture birds’ attention [5].

Considerable evidence has shown that the avian midbrain attention network performs saliency mapping, and space-specific deficits in visual orientation discrimination can be caused by lesions in the midbrain attention network [4,6,7,8,9]. The midbrain network consists of optic tectum (OT) and isthmic nuclei [10,11], and further reversible inactivation research shows that the isthmi pars magnocellularis (Imc) implements a “winner-take-all” function in this network [12], playing a decisive role in visual salient object computing [13,14,15]. The more forcefully one object can excite the Imc neuron, the more likely it can obtain access to the working memory [16], by diffusing robust inhibition to other competitors spreading over the visual space [14,15].

In addition, previous studies concerning Imc showed that objects moving in different directions induced unsymmetrical response strengths [17,18], implying differential saliency for objects moving in different motion directions, but the underlying physiological basis of this directional preference is unclear. Moreover, related anatomical studies have shown that OT neurons in the dorsoventral column arrange their receptive fields (RFs) perpendicular to the horizontal meridian; thus, the RF of Imc neurons receiving above the tectal afferents is vertically elongated in visual space [10,18,19]. Considering the fact that extensive research has also reported the relationship between RF structure and stimulus selectivity [20,21,22,23,24,25], we thus wanted to know if the Imc’s asymmetrical response for individual direction resulted from its special RF structure, as well as how the latter contributes to Imc’s directional preference.

Furthermore, the construction of neural computational models can provide help in understanding the neural mechanisms responsible for the observed phenomenon. An active area of research has attempted to build biologically plausible models that can explain neuronal responses to different stimuli [26,27,28,29], and a branch of the most successful computational models to date are for neurons at the early stages of the visual pathway [30,31,32,33]. However, for research on the avian visual system, neural computational models that are based on existing physiological evidence and can well reproduce the directional tuning curve of Imc are extremely rare.

To investigate Imc’s directional tuning characteristics and its relationship with the special RF structure, in this study, two assumptions were introduced to be verified: (1) Imc’s significant response to preferred motion directions results from different dynamic response characteristics; (2) the response strength for motion in each direction benefits from the RF structure. Together, we predict that above hypotheses can be confirmed, and the insights gained in this study will deepen our understanding of the motion processing in early stages of avians’ visual system.

## 2. Materials and Methods

### 2.1. Animal Care and Approval

Neuronal recordings of Imc units were performed in 11 pigeons (*Columba livia*, 6 adult male pigeons and 5 adult female pigeons, 350–450 g body weight), which were housed in separate wire mesh cages under a 12:12 h dark-light cycle, with free access to cereal and water. All our experiments were conducted in accordance with the Animals Act, 2006 (China) for the use and care of laboratory animals, and were approved by the Animal Care and Use Committee of Zhengzhou University (No SYXK 2019-0002).

### 2.2. Surgery and Recordings

All experiments were conducted following protocols that have been described previously [34]. Briefly, surgery was started after the pigeons were anesthetized with 20% urethane (10 mL/kg body weight). After the birds closed their eyes and no longer responded to painful or auditory stimulation, they were transferred to a stereotaxic device (model ST-5ND-B; Chengdu Instrument, Chengdu, China). Their heads were then placed in a stereotaxic holder, and the right eye was kept open by retraction of the eyelid during the experiment, while the left eye was covered avoiding surrounding light. A small hole was then drilled into the bone to expose the left dorsal brain, allowing access to the Imc. Then, a small slit was made in the dura with a syringe needle, permitting dorsoventral penetration to the Imc. Throughout the experiment, the animal’s body temperature was maintained at approximately 41 °C with a heating panel.

Multi-unit activity was recorded under anesthesia using platinum–iridium metal multi-electrode arrays (impedance = 20–50 kΩ; Clunbury Scientific, Bloomfield Hills, MI, USA), which were inserted into the Imc using a micromanipulator. The Imc was targeted according to stereotactic coordinates for Imc (AP: 1.25–4.25 mm ML: 4–6 mm DV: 4–7 mm) based on pigeon’s brain atlas [35], and with reference to the activity pattern and characteristic RF of the individual unit. Dorsoventral penetration through the Imc was made at a medial-leading angle of 5° from the vertical angle to avoid the major blood vessel in the path to the Imc, and Imc targeting was validated at the outset of this study through anatomical lesions [36].

Local field potential signals recorded were amplified (4000×), filtered (0–250 Hz), and continuously sampled at 2 kHz using a Cerebus^®^ recording system (Blackrock Microsystems, Salt Lake City, UT, USA). The spike signals of units were recorded with 30 kHz sampling frequency, and extracted with a band-pass filter (250 Hz–5000 Hz). All neural data recorded were analyzed off-line using our customized MATLAB R2018a (The MathWorks) applications.

### 2.3. Visual Stimuli

Visual stimuli used in this study were generated using the MATLAB-based Psychophysics Toolbox (Psychtoolbox-3; www.psychtoolbox.org, accessed on 10 January 2019) and was run using Windows 10. The stimuli-presenting system was synchronized with a recording system. A 55-inch LED monitor (Philips 558M1RY; monitor size: 1209.6 × 680.4 mm; 3840 × 2160 pixels; running at 100 Hz) was placed 40 cm away from and tangential to the pigeon’s right eye to present monocular visual stimuli [18]. The luminance of the gray screen background was 118 cd/m^2^ while the black stimuli was 0.2 cd/m^2^ (measured with a luminance meter, TES-137; TES Electrical Electronic, Taipei, China).

Three visual stimuli were used for our data collection. The first one was a black dot (1.3°, 0.2 cd/m^2^), moving randomly along a series of parallel paths covering the whole screen against a gray background (118 cd/m^2^) at 20–40°/s, which was used to map the RF of the Imc neurons. This stimulus was continuously repeated for five trials, and each trial followed different pseudorandom order. To maximize the use of the presenting area, the position and angle of the monitor were adjusted to ensure that its long axis was parallel with the major axis of the Imc RF. The second visual stimulus, with the same dot (1.3°, 0.2 cd/m^2^) as described above, moving across the center of Imc RF in 16 directions (temporal-nasal direction was set as 0°) spaced by 22.5°, at 30°/s against a gray background (118 cd/m^2^), was used to measure the directional tuning curves of the Imc units. Each motion direction was repeated for five trials, and were randomized so that different motion directions were presented in a random order over the entire sequence of 80 sweeps. Each sweep was followed by a 200 ms inter-trial interval. In addition, the distances of motion in each direction were equal (60°) and then each sweep was presented for 2 s. The last visual stimulus was also used to measure the directional tuning curves of the Imc units, which was almost the same with the second one, except that the speed was adjusted to 60°/s (stimulus duration was 1 s).

### 2.4. Analysis

The experimental data presented here were acquired from the Imc single- and multi-units in 11 pigeons. Both local field potential and spike signals were stored simultaneously throughout the procedures. The “Wave_clus” spike-sorting toolbox [37], a fast and unsupervised algorithm for spike detection and sorting, was used to sort the recorded data into single units. We include only those units for analysis that have less than 5% of the spikes within 1.5 ms of each other (ISI criterion) [36].

The receptive fields (RFs) of the Imc units were estimated by calculating the response for each site on the screen. The analysis window was from 50 ms to 60 ms after the onset of the stimulus, and the baseline response was estimated during the 1 s before the stimulus onset. Neural responses were quantified as the firing rate in the analysis window minus that of baseline activity, and were averaged across all trials. Then, the response strength for each unit was normalized by its own maximum. The 2D grid of neuronal firing rates to the stimuli was analyzed by fitting the response with the following 2D Gaussian model:

(1) $$\begin{array}{r} f\left(x,y\right)=\exp(-\frac{{\left(\left(x-x_{0}\right)\cos\theta -\left(y-y_{0}\right)\sin\theta \right)}^{2}}{2\sigma_{x}^{2}}\\ -\frac{{\left(\left(x-x_{0}\right)\sin\theta -\left(y-y_{0}\right)\cos\theta \right)}^{2}}{2\sigma_{y}^{2}}) \end{array}$$

where *f*(*x*, *y*) reflects the response to the stimuli at spatial position (*x*, *y*), site (*x*_0_, *y*_0_) being the center of the fitting model, *θ* being the orientation of its major elliptical axis relative to 0° in the physical space coordinate system, and *σ_x_* and *σ_y_* being the standard deviations (SDs) of the two axes. The fitting was performed by minimizing the least mean square error between the model and data. The RF center was determined as the center of contour of the model at 5% of the maximal response, and the size of the RF was the major and minor axis of the contour.

To measure directional tuning curves, the mean firing rate for each direction from stimuli onset to 200 ms after the finish was calculated and averaged across trials. Because firing rates evoked by identical stimuli were different from cell to cell, the mean firing rates were then normalized across all directions for each Imc unit. Directional tuning curves were smoothed for display purposes (by spline interpolation). To investigate the dynamic response characteristics of population Imc units to objects moving in individual directions, post-stimulus time histograms (PSTHs) were acquired from stimuli onset to 200 ms after the finish with 20 ms bins. For each unit, PSTHs for all directions were normalized by the maximum, and population mean PSTHs for each direction were determined as the average across the population Imc units. The response duration was defined as the time interval where responses of Imc units exceeded 5% of the maximal instantaneous firing rate of that sweep.

To test the significance of Imc units’ directional preference, we thus introduced the concept of directional preference index (*DPI*), written as Equation (2):

(2) $$DPI_{i}=\frac{R_{i}-R_{null}}{R_{i}}$$

where *R_i_* denotes the mean response to the direction *i* and *R_null_* the mean response to “null” direction (nasal-temporal) [17]. Any direction with a *DPI* greater than 0.7 was called the preferred direction of that Imc unit, and Imc units with more than one preferred directions were labeled as significantly directional preference units.

To determine in detail whether the preferences for v-d and d-v motions benefited from long response durations or high firing rates, we used the PSTHs of the Imc population units in four orthogonal (n-t, v-d, t-n and d-v) directions. For quantitative and intuitive comparisons between the population Imc unit response characteristics of the above four directions, their maximal instantaneous firing rates were plotted against the response durations of these four directions. We performed correlation analysis to compare above three curves, and a Holm-Bonferroni multiple-comparison correction [38] was applied to adjusted *p*-values for the multiple-tests.

### 2.5. Model

We assumed that the response strength of Imc neuron is a linear summation of activated subregions, which fairly distribute within the RF. Therefore, response strength for object moving in each direction was linearly related to the intercept between the motion path and RF. To check if this mechanism could account for the present results, a neural computational modeling approach was used to predict the directional tuning curves of the Imc.

The RF of the Imc unit was modeled with an ellipse, whose center represented the RF center. The ellipse is written as follows:

(3) $$\frac{x^{2}}{L_{minor}^{2}}+\frac{y^{2}}{L_{major}^{2}}\leq 1$$

where (*x*, *y*) are the coordinates of points within the visual space. Considering the directional tuning curve to be simulated for an individual Imc unit was separately normalized, the parameters *L_minor_* and *L_major_* in Equation (3) thus merely represent length of the semi-minor and semi-major axis of RF model, respectively, but not the physical length of an Imc unit. Therefore, *L_minor_* was set as 1 in the following modeling. The directional motion path is described as follows:

(4) $$x=\left\{ \begin{array}{l} 0,\alpha =90{}^{\circ}\\ \frac{y}{\tan\alpha }+x_{0},\alpha \neq 90{}^{\circ} \end{array}\right.$$

where (*x*_0_, *y*_0_) are the coordinates of center of RF, while *α* is the anticlockwise angle relative to 0° in the visual space. Hence, the distance within the RF can be written as Equation (5):

(5) $$d=\left\{ \begin{array}{l} 2L_{major},\alpha =90{}^{\circ}\\ 2\sqrt{L^{2}{}_{major}+\frac{1-L^{2}{}_{major}}{1+L^{2}{}_{major}\tan^{2}\alpha }},\alpha \neq 90{}^{\circ} \end{array}\right.$$

where *α* has the same meaning as in Equation (4), *θ*, *σ_x_*, and *σ_y_* are defined in Equation (1). Therefore, the predicted response strength is calculated as follows:

(6) $$R_{predict}=W\frac{4\sigma_{x}^{}\sigma_{y}^{}}{\sqrt{2\sigma_{x}^{2}\sin^{2}\left(90-\theta +\alpha \right)+2\sigma_{y}^{2}\cos^{2}\left(90-\theta +\alpha \right)}}+B$$

where *R_predict_* indicates the predicted response strength, *W* denotes the weight, and *B* represents the bias; other variables has been defined in the above Equations. To simulate the “null” direction around 0°, the following parabolic function was introduced:

(7) $$lx-my^{2}=1$$

where *l* and *m* are the free parameters to be fitted.

Therefore, the RF of the Imc unit was modeled with the Equation (3). An oblique line going through the center of the RF with an included angle (from 22.5° to 360° separated by 22.5°) relative to the n-t direction modeled by Equation (4), was used to simulate the path of dots moving in each direction. The distance of each path within the Imc RF was calculated using Equation (5). While the major axis of RF recorded in our experiment was vertical, the predicted response strength could be represented by Equation (6). In addition, a “gap” in the RF customized for motion directions near the n-t direction, modeled with Equation (7), was included to the modified model.

Finally, an object function was built as the mean square error of differences between predicted results and actual data. Thus, the parameter-solving process was transformed into an optimization problem, making the mean square error between the predicted and actual data the minimum. To solve the above nonlinear optimization problem, we used the “*GlobalSearch*” algorithm [39] in MATLAB to obtain optimal parameters for the neural computation model. The “*MaxIterations*” and “*MaxFunEvals*” criteria in the options of optimization problem were set to 10^3^ and the termination tolerance was then set to 10^−6^.

Although our neural computational model may predict the preference for v-d and d-v motion directions and even the total directional tuning curves at both speeds, to demonstrate that if Imc’s directional preference resulted from the special RF structure, the actual RF structures (ratio of the major axis versus minor axis) and predicted ones for each Imc unit should be compared.

## 3. Results

A total of 67 units were recorded in our experiment, and 2 units presenting unstable responses to repeat stimuli were discarded. As a result, the remaining 65 units were used for following analyses in this study.

### 3.1. RF Mapping and Directional Preferences within the Population Imc Units

**Figure 1:**
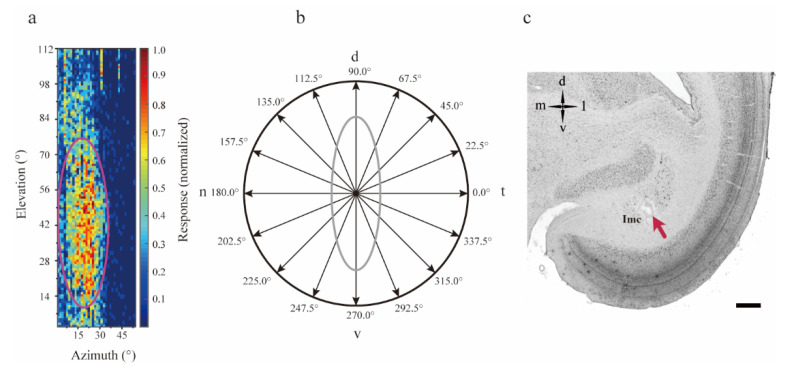
The special structure of the isthmi pars magnocellularis (Imc) receptive field. (**a**) A two-dimensional grid shows an example Imc unit response strength to stimuli in visual space, while the purple ellipse represents the receptive field (RF, range approximately 30° horizontal and 75° vertical) fitted by a two-dimensional Gaussian model. The strength was normalized by the maximum. (**b**) A dot moving across the RF center in 16 directions spaced by 22.5°, was used to measure the Imc directional tuning curve, and the n-t direction was set as 0°. The gray oval represents the fitted RF. (**c**) Bright field photomicrograph of 40 μm Nissl-stained coronal section, the red arrow indicates the electrolytic lesioned recording site of a sample unit. Scale bar-500 μm. d—dorsal; n—nasal; t—temporal; v—ventral.

The example Imc unit we recorded exhibited a special elongated RF (Figure 1a), which agreed with the results of a previous study. The RF center of this unit was measured as described above, and the directional tuning curve was evaluated with dots moving across the center in 16 directions spaced by 22.5°, at 30°/s and 60°/s (Figure 1b). After data recording was finished, the actual site of the recorded Imc unit in the brain was confirmed (Figure 1c).

**Figure 2:**
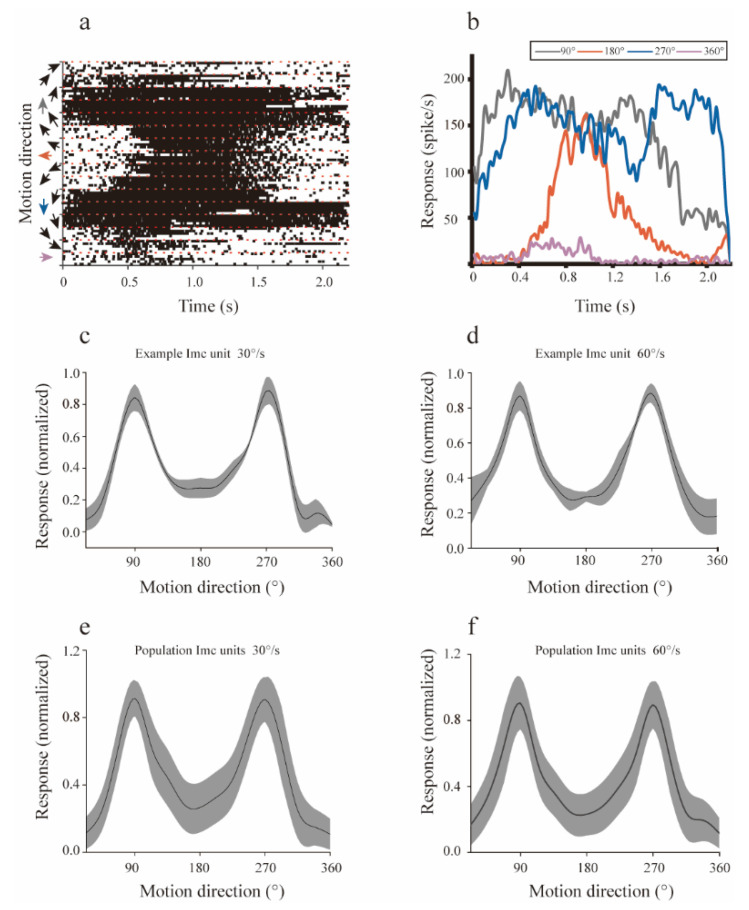
The directional tuning curve of the isthmi pars magnocellularis (Imc). (**a**) Spike raster plots of an example Imc unit to motion at 30°/s in 16 directions, and each direction was repeated for five trials. (**b**) Post-stimulus time histograms (PSTHs) of an example Imc unit to dots moving at 30°/s in the ventral-dorsal (90°), dorsal-ventral (270°), temporal-nasal (180°), and nasal-temporal (360°/0°) directions. (**c**,**d**) The directional tuning curve for the example Imc unit measured at 30°/s and 60°/s, respectively. Each motion direction was repeated in five trials, and the response strength for each direction was averaged across trials. The black line represents the mean response strength, while the width of the shadow indicates the standard deviation. Directional tuning curves were smoothed for display purposes (by spline interpolation). (**e**,**f**) The mean directional tuning curve of the population Imc units measured at 30°/s and 60°/s, respectively. Each motion direction was repeated in five trials, and the response strength for each direction was averaged across trials. The same format as (**c**).

Intuitively, the number of spikes emitted by the example Imc unit to different directions varied greatly (Figure 2a) and the PSTHs showed a huge difference in the dynamic response of the example Imc unit to the four motion directions (Figure 2b). Then, the mean firing rate was calculated to quantitate the response strength for each direction. The example unit showed asymmetrical response strengths for individual motion directions at 30°/s (Figure 2c). This Imc unit especially preferred the dots moving in d-v and v-d directions. This case was similar to the directional tuning test at 60°/s (Figure 2d), which meant that the dots moving in d-v and v-d directions were most salient for that example Imc unit.

To determine the universality of the motion directional preference in population Imc units, we evaluated the directional tuning curves of all 65 recorded units at 30°/s and 60°/s. To dots moving at a speed of 30°/s, the population Imc units we recorded showed similar directional preferences as the example unit (Figure 2e), and the most salient stimuli for the population Imc units were dots moving v-d and d-v across their RFs, with a weak salience for temporal-nasal (t-n) motion and little (if any) for nasal-temporal (n-t) motion. Moving speed did not change this trend (Figure 2f). Thus, the Imc’s preference for v-d and d-v directions may not be dependent on the moving speed, but was primarily, if not exclusively, tuned by motion directions.

Overall, 89% (58/65) Imc units we recorded showed directional preference, and the two directions that the greatest proportions of Imc units preferred were d-v and v-d directions (the proportions were 96.55% and 93.10%, respectively, see Table 1).

### 3.2. The Connection between Imc’s Directional Preferences and Dynamic Response Characteristics

Although the directional preference has been shown to be widespread in the Imc units, the underlying connection between Imc directional tuning and the dynamic response characteristics of each motion direction is unclear.

**Figure 3:**
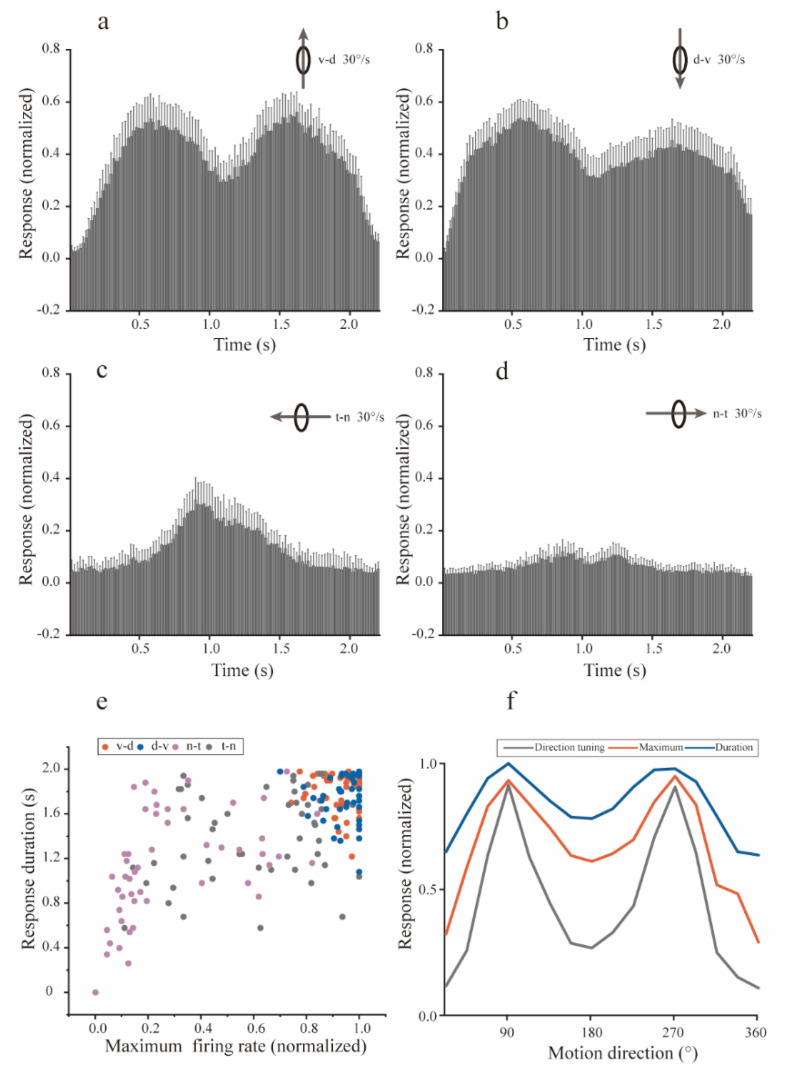
Dynamic response characteristics of isthmi pars magnocellularis (Imc) units to motion at 30°/s. (**a**–**d**) Population Imc units’ post-stimulus time histograms (PSTHs) to dots moving at 30°/s in the ventral-dorsal, dorsal-ventral, temporal-nasal, and nasal-temporal directions. The PSTH for each Imc unit was normalized to the respective maximum, and the mean PSTHs shown here are the mean dynamic responses for the direction v-d direction, from the stimuli onset to 200 ms after the finish. Error bars show 95% confidence intervals. (**e**) Scatterplot of population Imc units’ response characteristics. The response duration was plotted against the maximal firing rate, for each Imc unit to motion in the ventral-dorsal (red dot), dorsal-ventral (blue dot), nasal-temporal (purple dot), and temporal-nasal (gray dot) directions at 30°/s. (**f**) Plots of directional tuning (gray curve), maximal instantaneous firing rate (orange curve), and response duration (blue curve), normalized by respective maxima for population Imc units, suggesting close relationships with each other. d—dorsal; n—nasal; t—temporal; v—ventral.

The PSTHs of four orthogonal (n-t, v-d, t-n, and d-v) directions showed that moving objects induced robust responses (Figure 3a–d), especially for the v-d and d-v motion directions. The dots moving v-d and d-v induced significantly strong and long-lasting responses (Figure 3e), with shorter durations and weaker responses for the t-n and n-t directions. To determine possible correlations between the response strength of 16 motion directions and the dynamic response characteristics (Figure 3f), we performed correlation analyses of the population mean directional tuning with response durations, as well as with the maximal firing rates. The directional tuning of the Imc unit was closely related with the largest instantaneous firing rate (Table 2, Spearman’s correlations with Holm-Bonferroni correction, r = 0.99, *p* < 0.05), as well as the response durations of each direction (Spearman’s correlations with Holm-Bonferroni correction, r = 0.97, *p* < 0.05). Furthermore, there was a strong association between the durations and firing rates (Spearman’s correlations with Holm-Bonferroni correction, r = 0.96, *p* < 0.05).

**Figure 4:**
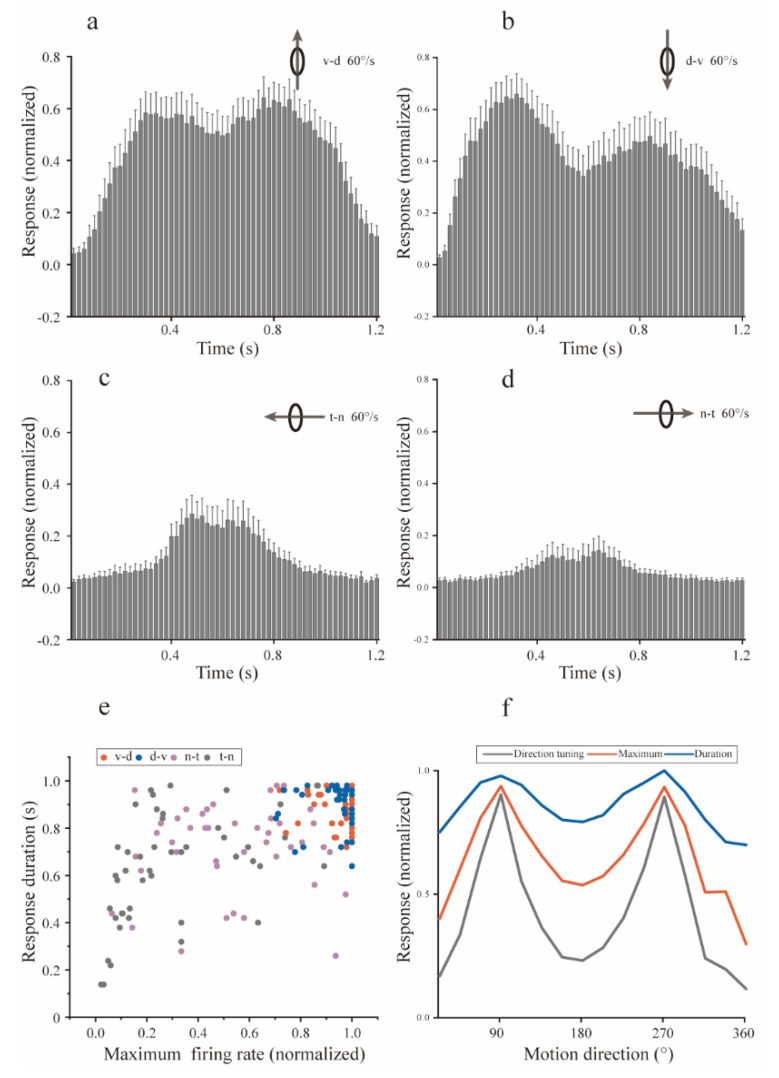
Dynamic response characteristics of isthmi pars magnocellularis (Imc) units to motion at 60°/s. (**a**–**d**) Population Imc unit post-stimulus time histograms (PSTHs) to dots moving at 60°/s in the ventral–dorsal, dorsal–ventral, temporal–nasal, and nasal–temporal directions. PSTH for each Imc unit was also normalized using the respective maxima, and the mean PSTHs showed the mean dynamic response for the v-d direction, from the onset of stimuli to 200 ms after the finish. Error bars show 95% confidence intervals. (**e**) Scatterplot of population Imc units’ response characteristics. The response duration was plotted against the maximal firing rate, for each Imc unit to motions in the ventral-dorsal (red dot), dorsal-ventral (blue dot), nasal-temporal (purple dot), and temporal-nasal (gray dot) directions at 60°/s, respectively. (**f**) Plot of directional tuning, maximal instantaneous firing rate, and response duration, normalized to the respective maximum for each population of Imc units, which shows a close relationship with each other. d—dorsal; n—nasal; t—temporal; v—ventral.

For dots moving at 60°/s, the same analysis was also conducted to investigate possible relationships between the directional tuning and the Imc dynamic response characteristics. The d-v and v-d motions were also most salient for the population Imc units (Figure 4a,b), while dots moving in the n-t and t-n directions weakly excited the Imc (Figure 4c,d). Statistical analyses also showed that response strengths for the above directions benefited from the response durations and maximum instantaneous firing rates (Figure 4e,f). Correction analyses (Table 2, right panel) showed that the mean directional tuning of the Imc population was closely associated with the population response duration (Spearman’s correlations with Holm-Bonferroni correction, r = 0.99, *p* < 0.05), which was the case for directional tuning and the maximal firing rate (Spearman’s correlations with Holm-Bonferroni correction, r = 0.99, *p* < 0.05) at 60°/s. In addition, the response durations of the population Imc units showed similar relationships with the largest instantaneous firing rates (Spearman’s correlations with Holm-Bonferroni correction, r = 0.97, *p* < 0.05) at 60°/s and 30°/s.

At different moving speeds, the preferences for d-v and v-d motion directions were similar, and the connection between Imc’s directional preferences and dynamic response characteristics were almost the same. Regarding objects moving in the v-d and d-v directions, the significant response strength resulted from both the long response duration and the high maximal instantaneous firing rate. Hence, the neural basis underlying this motion directional preference should be able to simultaneously improve the Imc response strength and duration.

### 3.3. A Neural Computational Model Predicting the Directional Preference of the Imc

Based on the abovementioned analyses and previous anatomical results [10,18,19], we assumed that the distance of the motion path within the Imc RF (see Section 2) may be the most likely option, from which the preference for motion directions could benefit.

**Figure 5:**
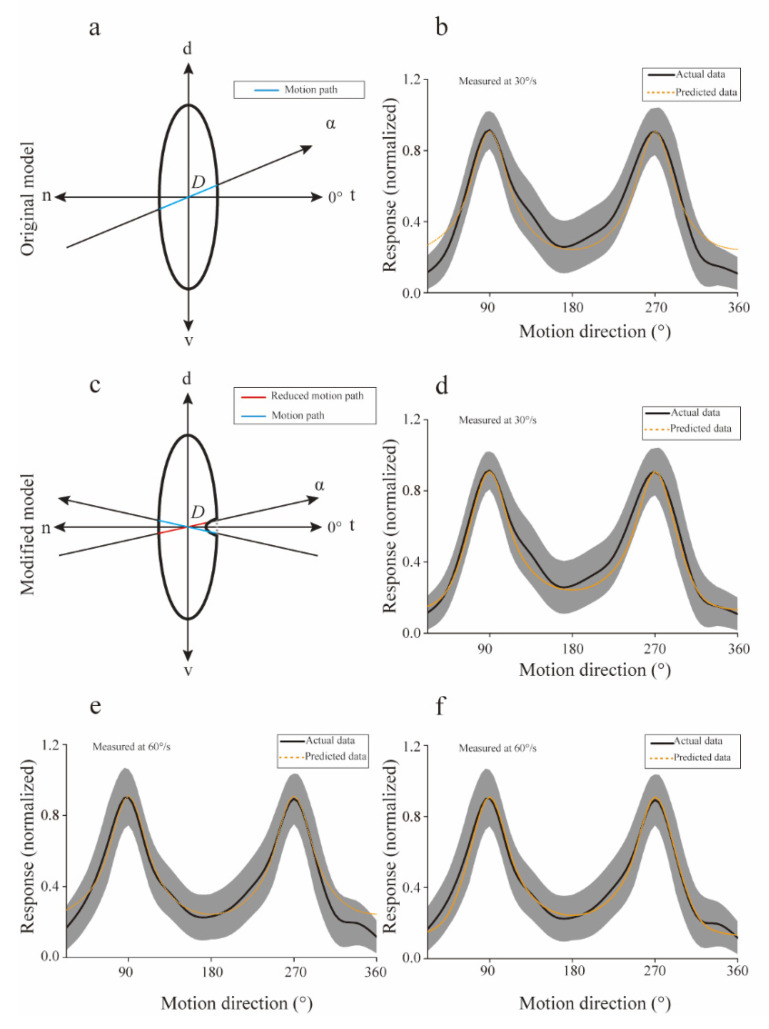
A computational model introduced to predict the preference for motion directions. (**a**) The receptive field of isthmi pars magnocellularis (Imc) units was modeled with an ellipse, while the moving path was simulated by an oblique line (α denotes the counterclockwise angle from nasal-temporal) going through the receptive field (RF) center. We assumed the response strength for each direction was linearly related with the intercept, D. (**b**) Predicted (orange) directional tuning curve followed the actual directional tuning (black) curve at 30°/s (Mean square error = 0.007). The width of the shadow indicates the standard deviation of actual data. (**c**) The modified model was added with a gap to simulate the “null” direction around the n-t direction. Thus, the motion path was reduced around n-t direction (such as the red path), but had no effect on other directions (such as the blue path). (**d**) The predicted (orange) directional tuning curve followed the actual directional tuning (black) curve at 30°/s (mean square error = 0.003). (**e**) The original model predicts the directional tuning curve (orange), when compared with the actual directional tuning (black) curve at 60°/s (mean square error = 0.003). (**f**) The modified model predicted the directional tuning curve (orange), when compared with the actual directional tuning (black) curve at 60°/s (mean square error = 0.001). d—dorsal; n—nasal; t—temporal; v—ventral.

The computational model (Figure 5a) described in Materials and Methods accurately predicted the directional tuning curves (Figure 5b, Mean square error = 0.007), especially the salient motion directions (v-d and d-v). These results suggested that the preferences for v-d and d-v directions may include linear correlation with the motion distance within the RF at 30°/s. However, the simulated results did not agree well with the actual data for directions around n-t direction, implying that a necessary component was omitted. The newly modified model (Figure 5c) more precisely (mean square error = 0.003) followed the real experimental data at all motion directions adopted in our study (Figure 5d).

Apart from the directional preference predictions for motion at 30°/s, our model was also used to predict the directional tuning of the population Imc units for motion at 60°/s. The simulation performance of the original model was also not fine (mean square error = 0.003) for motion at 60°/s (Figure 5e), but was significantly improved (mean square error = 0.001) after adding a “gap” in the RF for directions around n-t (Figure 5f). The above simulated results supported our assumption that the response of each motion direction was likely to be linearly related to the stimuli distance within the RF.

### 3.4. Directional Preference in Imc Benefits from Its Special Receptive Field Structure

**Figure 6:**
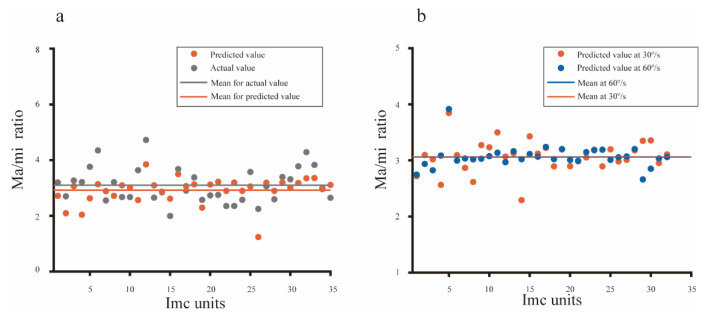
Physiological evidence supporting the biological plausibility of the computational model. (**a**) The structure predicted for each Imc unit was plotted against the actual data that was measured with an ellipse function, showing a good fitting performance (mean square error = 0.3662). (**b**) The receptive field structure predicted with data collected at 30°/s was very close (mean square error = 0.0723) to results predicted with data obtained at 60°/s from the same Imc unit, which implied the speed independence of directional tuning in Imc.

The computational model accurately predicted the mean directional tuning of population Imc units to dots moving at 30°/s and 60°/s (Figure 5d,f), and the simulated RF structures (ratio of major axis versus minor axis, 2.92 for 30°/s, 3.06 for 60°/s) were close with the actual data (ratio of major axis versus minor axis, 3.09 for 30°/s, 3.05 for 60°/s). In addition, the actual structure of an individual Imc unit was also calculated, and then compared with the predicted data. The actual ratio of the major axis versus minor axis was plotted against the predicted ratio for each Imc unit (Figure 6a), implying fine fitting performance (mean square error = 0.3662). Furthermore, for the same Imc unit, structures predicted by computational model optimized with data collected at 30°/s and 60°/s, respectively, were also compared. The simulated results agreed well with each other (Figure 6b, mean square error = 0.0723), which was in accordance with the actual physiology. Hence, these physiological results strongly supported our computational model, which meant that the preference of motion direction usually benefited from the stimuli distance within the RF. Thus, the avian species may have evolved a vertically elongated RF structure to generate the most saliency for the d-v and v-d motions.

## 4. Discussion

The experimental results suggested that the Imc units preferred objects moving in the v-d and d-v directions, which introduced a higher instantaneous firing rate and longer response duration. With the aid of a neural computational model, we were able to predict the preference for v-d and d-v motion directions, as well as the total directional tuning curve. Together, the results suggested that the Imc, which plays a key role in saliency computing and global competition, may has been “designed” with an elongated RF to capture objects moving in v-d and d-v directions. Overall, the results fit well with what we have predicted.

Almost all of the Imc units tested in our study exhibited oval-shaped field RFs, classical preference for some motion directions, and a clear “null” direction, from nasal to temporal in the visual space, which induced little, if any, response. All above results are consistent with previous research findings [17,18]. On the other hand, an index (*DPI*) was introduced to quantify the degree of Imc units’ significant selective tuning to motion directions; then statistical analysis for directional preference units and the preferred motion directions was performed in our study, showing that a large portion of Imc units preferred dorsal–ventral and ventral–dorsal motions; correlation analysis between directional preference and dynamic response characteristics was also tested, and these work were ignored in above literatures.

Compared with other directions, directional tuning for the n-t and t-n directions was not well predicted by the computational model. Our model was built on the assumption that subregions fairly distribute within the RF [40,41], meaning that the response induced by per unit length motion was the same (e.g., the density of the subunit of the Imc RF along an arbitrary direction through the RF center was equal). The model parameters contributing to the good fit to the saliency of d-v and v-d directions, leading to a poor fit for the t-n direction, suggested an asymmetric density of subunits along the vertical axis and horizontal axis of the Imc. This is possible when considering previous anatomical studies of the Imc [10,19], in which OT neurons projecting to the same Imc neurons were shown to distribute along the vertical axis, and their RFs vertically overlaid with each other, but without an overlay along the horizontal axis [18].

Regarding motion directions around the n-t direction, the results predicted by the original model were higher than the actual experimental data, which could be due to the OT-Imc feedforward circuit [10,18,19,42,43,44]. Previous studies have shown that most OT neurons showed fairly broad tuning curves with well-defined “null” directions [17,25,45,46]. We therefore believe that the original model’s poor fitting performance for directions around n-t could be attributed to “null” directions inherited from OT neurons. The “gap” that we added to the modified computational model, which may have simulated the “null” response in that direction, significantly improved the fitting performance of the model. Therefore, the improved simulation performance resulting from the “gap” in turn proved the rationality of results in the above literature.

Considering that the Imc has been shown in recent studies [14] to be the underlying nucleus endowing OT neurons with saliency mapping, and it can itself represent the salient degrees of moving objects [14,15,47]. In pigeons, dots moving in the v-d and d-v directions led to the robust Imc response, which could easily capture an organism’s attention using the midbrain attention network [8,15]. Thus, the Imc response strength actually represented the saliency of motion directions. A previous study reported that the Imc adapted to repetitive motion directions by decreasing the strength of its response to a low level, which faded after several minutes, showing plastic directional tuning [48]. Thus, unsymmetrical saliency mapping for individual motion directions, especially preferences for the v-d and d-v directions, could also be shaped by long-time, natural visual stimuli or predator–prey interaction [3,49,50]. This strong sensitivity of Imc neurons for upward and downward motion provides a typical example of functional neuronal adaptation to the specific visual environment [20,51,52,53,54,55].

Finally, our biologically plausible computational model accurately predicted the saliency mapping of motion directions prebuilt in Imc neurons, which provided a means to observe the avian accurate detection of immediate behavioral relevant objects [4,5,56]. The study also added new successful cases to the modeling of early stages of visual salient object processing [55,57,58,59]. Based on our results, future studies should further investigate Imc’s processing mechanisms for targets moving in different directions, for which a neural computational model that can reproduce the dynamic response characteristics of Imc neurons needs to be more finely developed.

Nevertheless, all studies have some limitations, including our study. Here, we present data have been collected with extracellular electrophysiological recordings within pigeon Imc, which locates in the early stages of the visual information processing, showing different response strength to varied motion directions. However, it is of interest for future studies to further investigate whether an object moving in an individual direction will induce different behavior feedback (e.g., different FID, escape strategy).

## 5. Conclusions

Our work here investigated the dynamic response characteristics of Imc units, implying that its directional preference not only results from the response duration but also the high instantaneous firing rate. Furthermore, we built a neural computational model, which well predicted the direction tuning curves, possessing parameters that agree with the actual receptive field structure. Thus, these results show that directional preference in Imc may be prebuilt within the special receptive field structure. Taking into account that repetitive stimuli will reshape the visual processing [48,60,61], the Imc’s receptive field structure may evolve along with long-term interaction with the natural visual stimuli, leading to ecological benefits for avians’ survival.

## Figures and Tables

**Table 1:** The number of directional preference units and statistical comparison of the preferred directions.

Significantly Directional Preference Units	58							
**Preferred direction**	**22.5°**	**45°**	**67.5°**	**90°**	**112.5°**	**135°**	**157.5°**	**180°**
Proportion	6.90%	51.72%	84.48%	93.10%	82.76%	70.69%	43.10%	41.38%
**Preferred direction**	**202.5°**	**225°**	**247.5°**	**270°**	**292.5°**	**315°**	**337.5°**	**360°**
Proportion	50.00%	67.24%	87.93%	96.55%	81.03%	39.66%	18.97%	0

**Table 2:** Correlation analysis of direction modulation curve, maximal firing rate, and response duration.

Motion at 30°/s				Motion at 60°/s			
Correlation	Direction tuning	Duration	Maximum	Correlation	Direction tuning	Duration	Maximum
Direction tuning	/	0.97 *	0.99 *	Direction tuning	/	0.99 *	0.99 *
Duration	0.97 *	/	0.96 *	Duration	0.99 *	/	0.97 *
Maximum	0.99 *	0.96 *	/	Maximum	0.99 *	0.97 *	/

* *p* < 0.05, with Holm-Bonferroni correction.

## Data Availability

The data that support the findings of this study are available on request from the corresponding author.
